# Is pericapsular nerve group block superior to other regional analgesia techniques following total hip arthroplasty? a systematic review and network meta-analysis

**DOI:** 10.1186/s13741-024-00455-y

**Published:** 2024-10-04

**Authors:** Lang Wan, Hua Huang, Fumin Zhang, Yanbing Li, Yantao Zhou

**Affiliations:** Department of Orthopedics, The Central Hospital of Enshi Tujia and Miao Autonomous Prefecture, Enshi, 445000 Hubei China

**Keywords:** Pain, Analgesia, Total hip arthroplasty, Systematic review, Meta-analysis

## Abstract

**Background:**

A systematic review and network meta-analysis (NMA) to compare the safety and efficacy of pericapsular nerve group block (PENGB) with other regional analgesia techniques in patients undergoing total hip arthroplasty (THA).

**Methods:**

We searched PubMed, Embase, Web of Science, and the Cochrane Library for relevant research from inception to May, 2024. Randomized controlled trials (RCTs) comparing PENGB with other regional analgesia techniques in patients undergoing THA were included. The primary outcome was resting pain scores at 6 h after surgery. The NMA was made by using Stata 15.1 software. Potential risk of bias was assessed by using CINeMA. Sensitivity and subgroup analyses were performed on the primary outcome.

**Results:**

A total of 11 RCTs including 766 patients were eligible for inclusion. For postoperative resting and movement pain scores within 24 h analysis, PENGB + periarticular local anesthetic infiltration (PLAI) was found to be significantly more effective than other treatments and its Surface under the cumulative ranking curve (SUCRA) was the lowest. Moreover, PENGB + PLAI was ranked the best in reducing opioid consumption within 24 h and the length of hospital stay. PENGB was found to have significantly lower incidence of quadriceps motor block and postoperative nausea and vomiting (PONV).

**Conclusions:**

PENGB is more likely to reduce the incidence of quadriceps motor block and PONV in patients undergoing THA, but PENGB + PLAI is superior to other regional analgesia techniques (PLAI, PENGB, fascia iliaca compartment block, and quadratus lumborum block) in improving postoperative pain and shortening the length of hospital stay.

**Trial registration number:**

CRD42024538421.

**Supplementary Information:**

The online version contains supplementary material available at 10.1186/s13741-024-00455-y.

## Introduction

Total hip arthroplasty (THA) is a widely performed surgical procedure aimed at alleviating pain and restoring function in patients suffering from hip joint pathologies, particularly hip fracture in elderly people (Haleem et al. 2023). As the demand for THA continues to rise due to an aging population and increasing prevalence of hip-related conditions, effective post-operative pain management has become a critical focus of orthopedic practice. However, insufficient pain relief may lead to disrupt sleep, stress, and impair the recovery (Arriaga et al. 2022). The guideline of Enhanced Recovery after Surgery (ERAS) recommends that optimizing analgesia not only enhances patient comfort but also facilitates early mobilization, reduces the risk of complications, and can potentially improve overall surgical outcomes. (Sameer et al. 2023). Regional analgesia techniques, including nerve blocks and local anesthetic infiltration, have gained prominence as effective alternatives or adjuncts to systemic opioid analgesia in the management of post-operative pain following THA (Reider et al. 2024). These techniques aim to reducing pain scores at rest and on movement, postoperative opioid consumption and complications, length of hospital stay and enhancing the recovery (Guay et al. 2017; Kim et al. 2022). Femoral nerve block (FNB) (Lin et al. 2021), lateral femoral cutaneous nerve block (LFCNB) (Yoo et al. 2024), periarticular local anesthetic infiltration (PLAI) (Bravo et al. 2023), and fascia iliaca compartment block (FICB) (Aliste et al. 2021) are commonly used analgesic techniques for THA, which attributed to their validity and simplicity. The operations of quadratus lumborum block (QLB) (Wang et al. 2023) and lumbar plexus block (LPB) (Bravo et al. 2020) are relatively complex. LPB and suprainguinal FICB have equivalent analgesic efficacy (Bravo et al. 2020). But FNB, LPB and FICB can result in quadriceps motor block of the surgical limb which can delay recovery and patient discharge(Lin et al. 2021; Bravo et al. 2020). Girón-Arango et al. reported an emerging regional analgesia technique named pericapsular nerve group block (PENGB) which preserves of quadriceps muscle strength (Girón-Arango et al. 2018). Although recent conventional meta-analysis showed that PENGB effectively decreased pain scores and opioid consumption within 24 h following THA when compared with a placebo group (Ke et al. 2024; She et al. 2024; Pai et al. 2024), it is unclear whether PENGB is superior to other regional analgesia techniques.

Hence, we conducted a network meta-analysis (NMA) to systematically evaluate the direct and indirect evidences to provide a reference basis for clinical practice. We aim to determine the relative efficacy of PENGB compared to other regional analgesia techniques after THA.

## Methods

The protocol was registered and published in PROSPERO database (CRD42024538421). This NMA adhered to the PRISMA for Network Meta-Analyses (Hutton et al. 2015).

### Eligibility criteria

We included studies according to PICOS criteria: patients undergoing THA (P); regional analgesia techniques included LPB, FICB, PENG, LFCN, PLAI, QLB, or a combination (I); one of the regional analgesia techniques (C); postoperative pain scores and opiates consumption within 24 h, PONV, quadriceps motor block, or length of hospital stay (O); RCTs (S). We excluded studies that they were met the following criteria: (1) incomplete and duplicate data; (2) parallel and crossover studies; (3) unpublished studies.

### Study selection

Two independent investigators (W.L. and H.H.) searched PubMed, Embase, Web of Science, and the Cochrane Library for relevant research from inception to May, 2024, with the key words including “pericapsular nerve group,” “total hip arthroplasty,” and “pain.” They reviewed all titles, abstracts, and then full texts. The disagreements on eligibility were resolved by the third reviewer (Z.F.M).

### Data extraction and data retrieval

Two authors (W.L. and H.H.) extracted the data independently. Disagreements were resolved by consensus. The relevant data from eligible studies includes the following: author, year of publication, country, blinding, ASA, type of anesthesia, treatments description, sample size, pain assessment methods, rescuing analgesic regimens, and outcomes. The primary outcome was postoperative resting Visual Analogue Scale (VAS) at 6 h. The secondary outcomes included resting and movement VAS within 24 h, opiates consumption within 24 h, postoperative nausea and vomiting (PONV) within 24 h, quadriceps motor block within 24 h, and length of hospital stay (LOS). All pain scores of eligible studies were converted to the 0—10 VAS. VAS at 6 h was the maximum value from 0 to 6 h after surgery, and so were the other endpoints of VAS. Opiates consumption was converted to intravenous morphine equivalent doses (mg).

### Certainty of evidence

The Cochrane Collaboration's tool and Confidence in Network Meta-analysis (CINeMA 2.0.0 version) were used to assess the certainty of evidence. Cochrane Collaboration's tool included random sequence generation, allocation concealment, performance bias, detection bias, attribution bias, reporting bias, and other biases (Higgins et al. 2011). The CINeMA included six domains: within-study bias, reporting bias, indirectness, imprecision, heterogeneity and incoherence (Nikolakopoulou et al. 2020).

### Statistical analysis

The NMA was carried out by utilizing STATA 15.1, the data were synthesized by random-effects model. For dichotomous outcomes, the pooled odds ratios (OR) and 95% confidential intervals (CIs) were calculated. For continuous data, the mean differences (MD) and 95% CIs were evaluated. For the data expressed as median and inter-quartile range, it was transformed to mean and standard deviation by using the earlier discussed methods (Luo et al. 2018; Wan et al. 2014). *P* value < 0.05 was defined as statistical significance.

Network geometry maps showed all direct comparisons; nodes of its corresponded to treatments; line thickness corresponded to the number of direct comparisons. A comparison-adjusted funnel plot was used to assess publication bias, which suggested publication bias if the symmetry around the zero line was affected. Forest plots were used to display study outputs. Node-splitting was used to test of local inconsistency. Netleague tables were used to show the relative effectiveness of each intervention. The Surface under the cumulative ranking curve (SUCRA) was used to estimate the ranking probabilities for all interventions, with lower values indicating superior effect in our NMA. Sensitivity analysis and subgroup analysis were performed for the primary outcome to explore the sources of heterogeneity.

## Results

### Study selection and characteristics

**Fig. 1:**
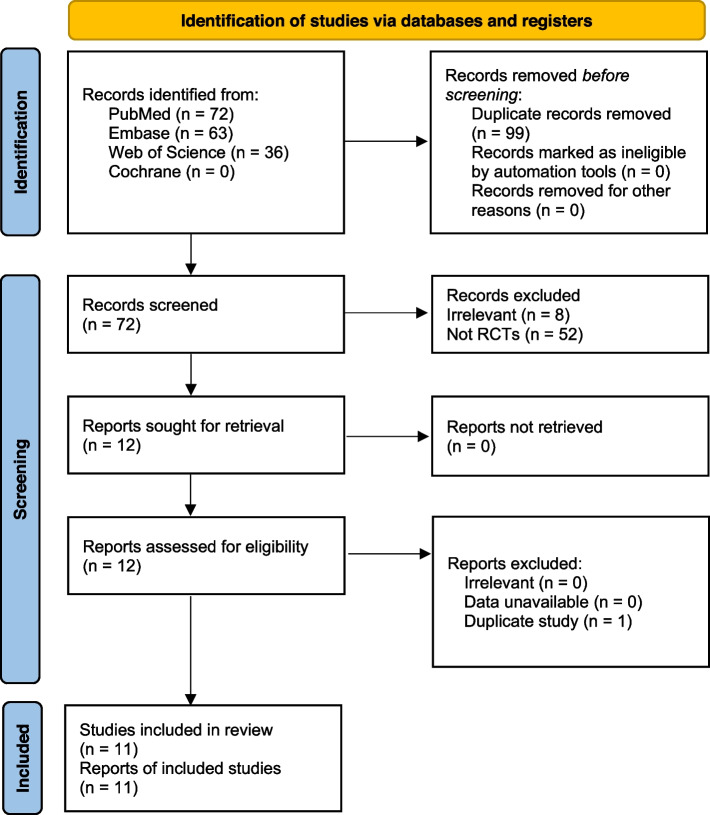
PRISMA diagram of study selection

We identified 72 potentially relevant records, 60 were excluded based on title and abstract alone, due to irrelevant intervention (*n* = 1), irrelevant control group (*n* = 7), or for not being an RCT (*n* = 52). we reviewed the full text of the remaining 12 potentially eligible studies, and excluded a duplicate study (*n* = 1). Consequently, a total of 11 RCTs (Bravo et al. 2023; Aliste et al. 2021; Wang et al. 2023; Lin et al. 2022; Zheng et al. 2022; Carella et al. 2023; Kong et al. 2022; Ye et al. 2023; Et et al. 2023; Hu et al. 2023; Liang et al. 2023) with 766 patients were included in this NMA and the analgesic techniques included FICB, PENG, PENG + LFCN, PENG + PLAI, PLAI, QLB. A flow diagram of literature inclusion was presented in Fig. 1. Table 1 showed the characteristics of included studies. 266 patients were assigned to PENGB and 500 to other regional analgesia techniques. The primary outcome was reported in 7 RCTs (Bravo et al. 2023; Aliste et al. 2021; Zheng et al. 2022; Carella et al. 2023; Kong et al. 2022; Et et al. 2023; Hu et al. 2023) with 444 patients. Using of PENGB and PLAI were the most frequent treatments followed closely by FICB. 5 trails (Wang et al. 2023; Kong et al. 2022; Ye et al. 2023; Hu et al. 2023; Liang et al. 2023) were in patients undergoing general anesthesia, 5 trails (Bravo et al. 2023; Aliste et al. 2021; Zheng et al. 2022; Carella et al. 2023; Et et al. 2023) were in patients undergoing spinal anesthesia. Table 2 showed a summary of evidence.

**Table 1:** Study characteristics of included studies

Study	Country	Blinding	ASA	Anesthesia method	Rescue analgesic regimen	Comparison (n)	Pain assessment method	Outcomes
Bravo et al. 2023	Chile	1	I-III	SA	A morphine PCIA + acetaminophen 1 g/6 h + ketoprofen 100 mg/8 h	PENG(30): 20 mL of bupivacaine 0.5% with epinephrine 5 µg/ mL	10-NRS	(1)(2)(3)(4)(5) (6)(7)(8)(9)(10)
						PLAI(30): 60 mL of bupivacaine 0.25% with epinephrine 5ug/mL and 30 mg of ketorolac		
Aliste et al. 2021	Chile	1	I-III	SA	A morphine PCIA + acetaminophen 1 g/6 h + ketoprofen 100 mg/8 h	PENG(20):20 ml ropivacaine 0.75%	10-NRS	(1)(2)(3)(4)(5) (6)(7)(8)(9)(10)
						FICB(20): 40 ml ropivacaine 0.375%		
Wang et al. 2023	China	2	I-III	GA	Celecoxib 200 mg/12 h.Morphine10 mg was injected if the patient was unable to tolerate the pain	PENG(45): 20 mL of ropivacaine 0.5% + 1:200,000 epinephrine	100-VAS	(4)(8)(9)
						QLB(45): 30 mL of ropivacaine 0.33% + 1:200,000 epinephrine		
Lin et al. 2022	Australia	2	I-IV	SA/GA	Acetaminophen and NSAIDs, even tramadol, oxycodone, and/or fentanyl	PLAI(30):100 mL of 0.1% ropivacaine with 1 mg epinephrine	10-NRS	(7)(9)
						PENG + PLAI(30): 20 mL of ropivacaine 0.5% for PENGB, 100 mL of 0.1% ropivacaine with 1 mg epinephrine for PLAI		
Zheng et al. 2022	Korea	1	II-III	SA	A fentanyl PCIA	PENG(25): 30 mL of ropivacaine 0.5%	10-NRS	(1)(2)(3)(7)(8)
						PLAI(27): 100 ml of ropivacaine 150 mg + ketorolac 60 mg + epinephrine 1 g		
Carella et al. 2023	Belgium	1	I-II	SA	A morphine PCIA. Paracetamol 1 g was injected at the end of surgery, oral paracetamol 1 g/6 h, and etoricoxib 60 mg/24 h	PENG(51):20 ml ropivacaine 0.75%	10-NRS	(1)(2)(3)(5)(6) (8)(10)
						FICB(51): 40 ml ropivacaine 0.375%		
Kong et al. 2022	China	2	II-III	GA	A fentanyl PCIA	PENG(25): 30 mL of ropivacaine 0.375%	10-VAS	(1)(3)(4)(6)(7) (8)(9)
						FICB(25): 30 mL of ropivacaine 0.375%		
Ye et al. 2023	China	2	I-III	GA	Celecoxib 200 mg/12 h.Morphine10 mg was injected if the patient was unable to tolerate the pain	PENG(40): 20 mL of ropivacaine 0.5% + 1:200,000 adrenaline	100-VAS	(4)(5)(7)(8)(10)
						PLAI(40): 30 ml of ropivacaine 0.33% + 1:200,000 adrenaline		
Et et al. 2023	Turkey	2	I-III	SA	IV paracetamol 1,000 mg/8 h and oral diclofenac 50 mg/8 h.Oral oxycodone 5 mg was administered if NRS score > 4	PENG(30): 20 ml of bupivacaine 0.5%	10-NRS	(1)(2)(3)(4)(5) (6)(8)(9)
						QLB(30): 30 ml of bupivacaine 0.5%		
Hu et al. 2023	China	2	I-III	GA	Celecoxib 200 mg/12 h.Morphine10 mg was injected if the patient was unable to tolerate the pain	PLAI(40): 40 mL of ropivacaine 0.5%	10-VAS	(1)(2)(3)(4)(5) (6)(7)(8)(10)
						PENG + PLAI(40): 20 mL of ropivacaine 0.5% for PENG block, 20 mL of ropivacaine 0.5% for PLAI		
Liang et al. 2023	China	1	II-III	GA	A tramadol + furbiprofenaxetil PCIA, and iv parecoxib sodium 40 mg/24 h	FICB(46): 30 ml ropivacaine 0.33%	10-VAS	(8)
						PENG + LFCN(46): 20 ml of ropivacaine 0.33% for PENG block, 10 ml of ropivacaine 0.33% for LFCN block		

*Abbreviations*: *SA* spinal anesthesia, *GA* general anesthesia, *PENGB* pericapsular nerve group block, *PLAI* periarticular local anesthetic infiltration, *QLB* quadratus lumborum block, *FICB* fascia iliaca compartment block, *LFCNB* lateral femoral cutaneous nerve block, *PCIA* patient-controlled intravenous analgesia, *VAS* visual analog scale, *NRS* numeric rating scale. (1) postoperative resting VAS at 6 h. (2) postoperative resting VAS at 12 h. (3) postoperative resting VAS at 24 h. (4) postoperative movement VAS at 6 h. (5) postoperative movement VAS at 12 h. (6) postoperative movement VAS at 24 h. (7) postoperative opioid consumption within 24 h (intravenous morphine equivalent, mg). (8) PONV, postoperative nausea and vomiting. (9) quadriceps motor block. (10) LOS, length of hospital stay (days)

**Table 2:** Summary of evidence

Outcomes	Patients (trails)	Results	Certainty (GRADE)
Resting VAS at 6 h after surgery	444 (7)	PENG + PLAI was superior to others	⨁⨁⨁⨁ high
Resting VAS at 12 h after surgery	394 (6)	PENG + PLAI was superior to others	⨁⨁⨁◯ moderate
Resting VAS at 24 h after surgery	444 (7)	PENG + PLAI was superior to others	⨁⨁⨁◯ moderate
Movement VAS at 6 h after surgery	460 (7)	PENG + PLAI was superior to others	⨁⨁⨁⨁ high
Movement VAS at 12 h after surgery	422 (6)	PENG + PLAI was superior to others	⨁⨁⨁◯ moderate
Movement VAS at 24 h after surgery	392 (6)	PENG + PLAI was superior to others	⨁⨁⨁◯ moderate
Postoperative 24-h cumulative opiates consumption	422 (7)	PENG + PLAI was superior to others	⨁⨁◯◯ low
PONV	706 (10)	PENG was superior to others	⨁⨁⨁◯ moderate
Quadriceps motor block	360 (6)	PENG was superior to others	⨁⨁⨁◯ moderate
Length of hospital stay	362 (5)	PENG + PLAI was superior to others	⨁⨁◯◯ low

### Assessment of *bias*

**Fig. 2:**
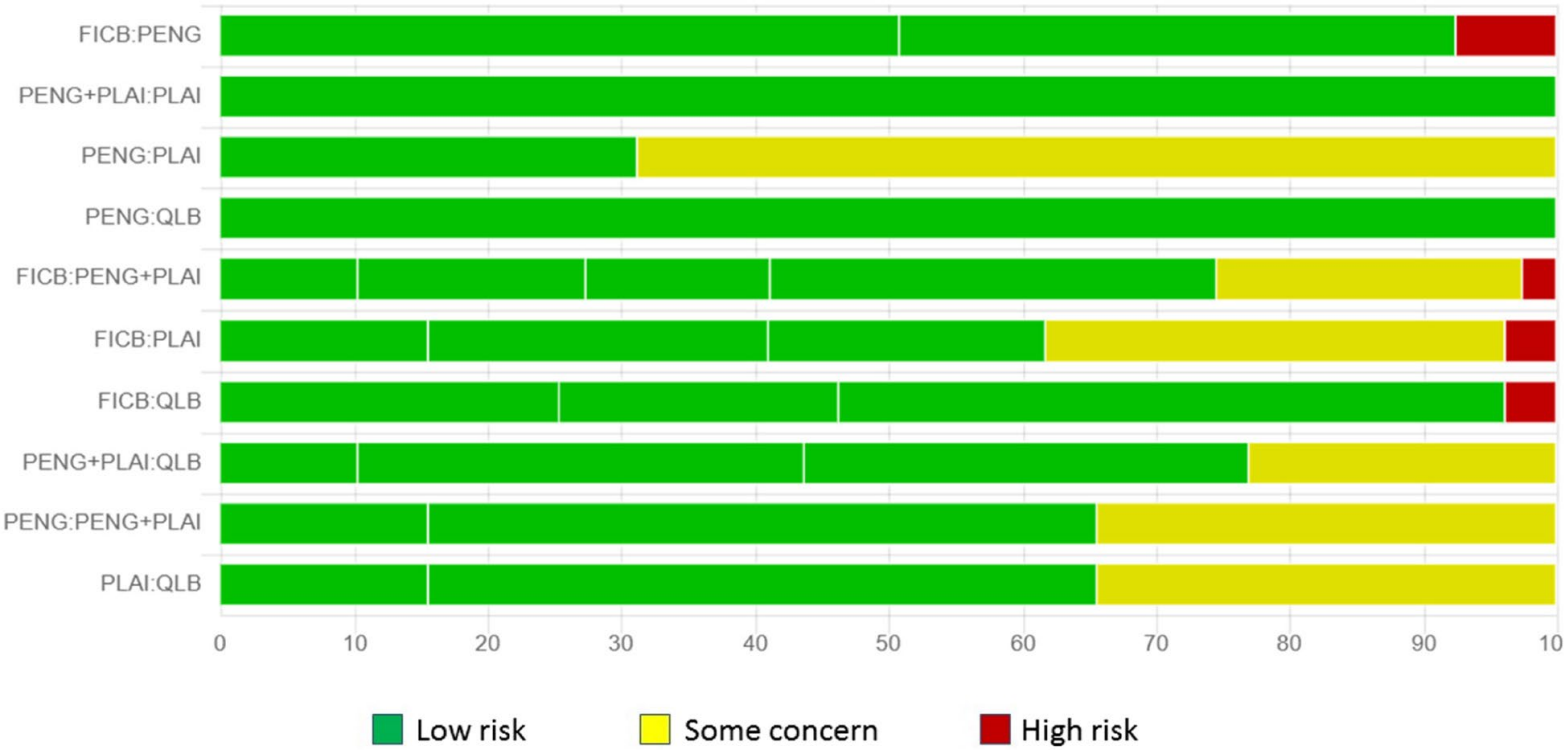
Risk of bias of resting VAS at 6 h after surgery

**Fig. 3:**
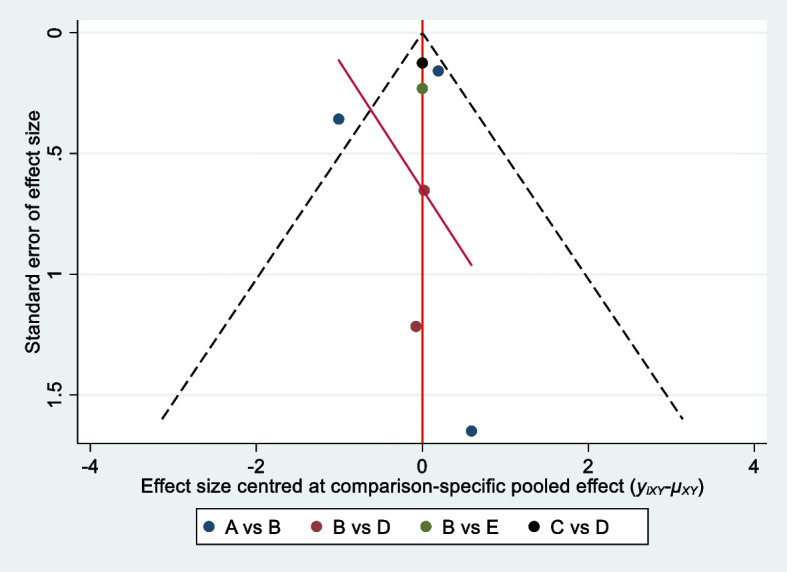
Funnel plot of postoperative resting VAS at 6 h

Risk of bias of the primary outcome is presented in Fig. 2. The funnel plot did not suggest any publication bias for the primary outcome (Fig. 3).

### Primary outcomes

#### Resting VAS at 6 h after surgery

**Fig. 4:**
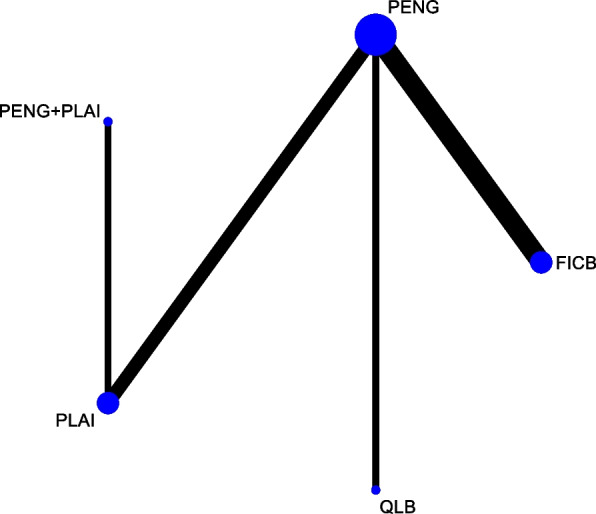
Network map of postoperative resting VAS at 6 h

**Fig. 5:**
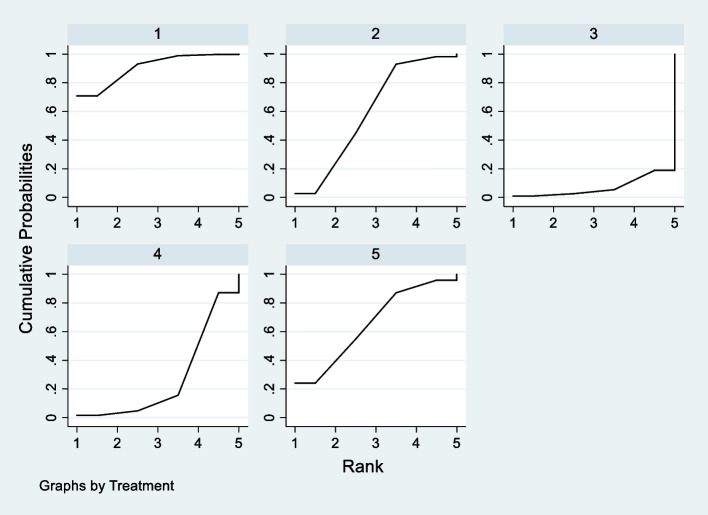
SUCRA of postoperative resting VAS at 6 h. 1 = FICB(90.6), 2 = PENG(59.7), 3 = PENG + PLAI(7.0), 4 = PLAI(27.2), 5 = QLB(65.5)

**Fig. 6:**
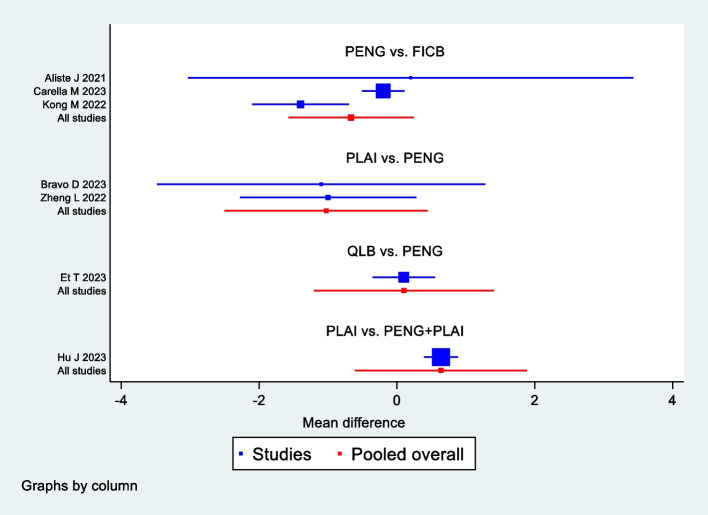
Forest plot of postoperative resting VAS at 6 h

Seven studies with 444 patients were included. The network map displayed complete, as all nodes could be connected (Fig. 4). The result of node-splitting did not show any significant inconsistency (Table 3). The SUCRA ranking indicated that PENG + PLAI attained the lowest value (7.0), followed closely by PLAI(27.2), PENG(59.7), QLB(65.5), FICB(90.6) (Fig. 5). The forest plot was showed in Fig. 6. Netleague tables of mixed estimates was showed in Table 4.

**Table 3:** Node-splitting of postoperative resting VAS at 6 h

Side	Direct		Indirect		Difference		P >|z|
	Coef	Std. Err	Coef	Std. Err	Coef	Std. Err	
FICB vs. PENG^a^	-.669429	.4636888	.9348291	14.65867	-1.604258	14.66601	0.913
PENG vs. PLAI^a^	-1.030412	.7541543	.9958844	17.57038	-2.026296	17.58661	0.908
PENG vs. QLB^a^	.0999999	.6661648	1.335966	56.62203	-1.235966	56.626	0.983
PENG + PLAI vs. PLAI^a^	.6399999	.6376629	-3.399068	35.47812	4.039068	35.48389	0.909

^a^ Warning: all the evidence about these contrasts comes from the trials which directly compare them

**Table 4:** Netleague of postoperative resting VAS at 6 h

	QLB	PLAI	PENG + PLAI	PENG
FICB	-0.57 (-2.16,1.02)	-1.69 (-3.43,0.04)	-2.33 (-4.47,-0.20)	-0.67 (-1.58,0.24)
	QLB	-1.13 (-3.10,0.84)	-1.77 (-4.10,0.57)	-0.10 (-1.41,1.21)
		PLAI	-0.64 (-1.89,0.61)	1.03 (-0.45,2.50)
			PENG + PLAI	1.67 (-0.27,3.60)
				PENG

### Secondary outcomes

#### Resting VAS at 12 h after surgery

Six studies with 394 patients were included. The network map displayed complete, as all nodes could be connected (Supplementary Fig. 1A). The result of node-splitting did not show any significant inconsistency (Supplementary Table 3A). The SUCRA ranking indicated that PENG + PLAI attained the lowest value (3.3), followed closely by QLB(42.9), PLAI(50.7), PENG(70.3), FICB(82.8) (Supplementary Fig. 5A). The forest plot was showed in Supplementary Fig. 2A. Netleague tables of mixed estimates was showed in Supplementary Fig. 4A.

#### Resting VAS at 24 h after surgery

Seven studies with 444 patients were included. The network map displayed complete, as all nodes could be connected (Supplementary Fig. 1B). The result of node-splitting did not show any significant inconsistency (Supplementary Table 3B). The SUCRA ranking indicated that PENG + PLAI attained the lowest value (11.2), followed closely by PLAI(40.4), QLB(52.7), PENG(60.9), FICB(84.6) (Supplementary Fig. 5B). The forest plot was showed in Supplementary Fig. 2B. Netleague tables of mixed estimates was showed in Supplementary Fig. 4B.

#### Movement VAS at 6 h after surgery

Seven studies with 460 patients were included. The network map displayed complete, as all nodes could be connected (Supplementary Fig. 1C). The result of node-splitting did not show any significant inconsistency (Supplementary Table 3C). The SUCRA ranking indicated that PENG + PLAI attained the lowest value (0.0), followed closely by PLAI(25.1), PENG(49.9), QLB(75.0), FICB(100.0) (Supplementary Fig. 5C). The forest plot was showed in Supplementary Fig. 2C. Netleague tables of mixed estimates was showed in Supplementary Fig. 4C.

#### Movement VAS at 12 h after surgery

Six studies with 422 patients were included. The network map displayed complete, as all nodes could be connected (Supplementary Fig. 1D). The result of node-splitting did not show any significant inconsistency (Supplementary Table 3D). The SUCRA ranking indicated that PENG + PLAI attained the lowest value (0.0), followed closely by PLAI(29.1), FICB(54.6), QLB(80.6), PENG(85.6) (Supplementary Fig. 5D). The forest plot was showed in Supplementary Fig. 2D. Netleague tables of mixed estimates was showed in Supplementary Fig. 4D.

#### Movement VAS at 24 h after surgery

Six studies with 392 patients were included. The network map displayed complete, as all nodes could be connected (Supplementary Fig. 1E). The result of node-splitting did not show any significant inconsistency (Supplementary Table 3E). The SUCRA ranking indicated that PENG + PLAI attained the lowest value (27.0), followed closely by QLB(39.5), PENG(44.0), PLAI(59.2), FICB(80.3) (Supplementary Fig. 5E). The forest plot was showed in Supplementary Fig. 2E. Netleague tables of mixed estimates was showed in Supplementary Fig. 4E.

#### Postoperative 24-h cumulative opiates consumption

Seven studies with 422 patients were included. The network map displayed complete, as all nodes could be connected (Supplementary Fig. 1F). The result of node-splitting did not show any significant inconsistency (Supplementary Table 3F). The SUCRA ranking indicated that PENG + PLAI attained the lowest value (15.1), followed closely by PENG(44.1), PLAI(48.6), FICB(92.3) (Supplementary Fig. 5F). The forest plot was showed in Supplementary Fig. 2F. Netleague tables of mixed estimates was showed in Supplementary Fig. 4F.

#### Postoperative nausea and vomiting (PONV)

Ten studies with 706 patients were included. The network map displayed complete, as all nodes could be connected (Supplementary Fig. 1G). The result of node-splitting did not show any significant inconsistency (Supplementary Table 3G). The SUCRA ranking indicated that PENG attained the lowest value (34.7), followed closely by FICB(45.5), PENG + LFCN(49.2), QLB(50.3), PLAI(52.2), PENG + PLAI(68.1) (Supplementary Fig. 5G). The forest plot was showed in Supplementary Fig. 2G. Netleague tables of mixed estimates was showed in Supplementary Fig. 4G.

#### Quadriceps motor block

Six studies with 360 patients were included. The network map displayed complete, as all nodes could be connected (Supplementary Fig. 1H). The result of node-splitting did not show any significant inconsistency (Supplementary Fig. 3H). The SUCRA ranking indicated that PENG attained the lowest value (26.8), followed closely by PLAI(33.8), PENG + PLAI(42.9), QLB(53.2), FICB(93.3) (Supplementary Fig. 5H). The forest plot was showed in Supplementary Fig. 2H. Netleague tables of mixed estimates was showed in Supplementary Fig. 4H.

#### Length of hospital stay(LOS)

Five studies with 362 patients were included. The network map displayed complete, as all nodes could be connected (Supplementary Fig. 1I). The result of node-splitting did not show any significant inconsistency (Supplementary Fig. 3I). The SUCRA ranking indicated that PENG + PLAI attained the lowest value (3.0), followed closely by FICB(61.6), PLAI(67.5), PENG(68.0) (Supplementary Fig. 5I). The forest plot was showed in Supplementary Fig. 2I. Netleague tables of mixed estimates was showed in Supplementary Fig. 4I.

#### Subgroup and sensitivity analysis

For the primary outcome, we performed subgroup analyses based on the type of anesthesia. The general anesthesia group included 2 trials with 130 patients, but the next analyses was prevented because the geometry map was disconnected (Supplementary Fig. 6E). The spinal anesthesia group involved 5 trials with 240 patients, the SUCRA ranking indicated that PLAI attained the lowest value (3.4), followed closely by PENG(47.2), QLB(66.8), FICB(82.5) (Supplementary Fig. 6B).

We attempted to make a sensitivity analysis by converting random effect model to fixed effect model, the original results did not reverse. We eliminated study one by one while ensuring the geometry map included all original comparisons, the new results determined the stability of conclusion.

## Discussion

Our NMA demonstrated that PENGB had lower incidence of quadriceps motor block and PONV. For postoperative resting and movement pain scores within 24 h analysis, PENGB + PLAI was found to be significantly more effective than other treatments (PLAI, PENGB, QLB, FICB) and its SUCRA was the lowest. Moreover, PENGB + PLAI was ranked the best in reducing opioid consumption within 24 h and the LOS. We speculated that PENGB covered the sensory innervation of anterior capsule of the hip joint and PLAI covered the anterior capsule and posterior compartment (Hu et al. 2023), so PENGB + PLAI more effectively inhibited of pain signal conduction from the periphery to the spinal cord at an early stage. Based on the type of anesthesia, the subgroup analyses showed that PLAI was more effective than other treatments (PENGB, QLB, FICB) in reducing resting VAS at 6 h. No major network inconsistency and heterogeneity were founded form the results of node-splitting and forest plots, the result of sensitivity analysis further indicated the stability of conclusion.

In recent meta-analysis, Farag et al. found that the efficiency of PENGB was comparable to FICB after hip fracture surgeries (Farag et al. 2023). Andrade et al. found that PENGB reduced opioid consumption within the first 24 h and reduced resting pain score at 12 h after hip fracture surgeries compared with the FICB (Andrade et al. 2023). The above meta-analysis only compared PENGB and FICB, but we evaluated all the treatments related to PENGB in THA. Hayashi et al. found that the PENGB might be superior to FICB and FNB for pain relief (Hayashi et al. 2024). These meta-analysis included all types of hip surgeries, which increased the heterogeneity of the results and decreased the reliability of the conclusions. These results were similar to ours, but we get a more reliable evidence of PENGB + PLAI with pain relief and PENGB with preservation of quadriceps muscle strength.

THA is one of the fast-effective surgeries to restore the function of hip joint after hip fracture (Descamps et al. 2023). Those patients usually experiences moderate to severe pain after surgery, which leads to anxiety and depression, prevents functional rehabilitation training, increases the risk of lower limb thrombosis and pneumonia, and prolongs the LOS (Descamps et al. 2023). Effective analgesia is helpful to muscle strength recovery and early activity, which reduces the risk of deep vein thrombosis, pulmonary embolism and pneumonia (Myles et al. 2024). regional analgesia techniques has become the basis of multimodal analgesia to control perioperative pain. Destruction of the hip joint capsular is the main source of pain after THA, regional analgesia techniques can be used for relief pain by blocking sensory nerves and branches.The femoral nerve (FN) and obturator nerve (ON) supplies the anterior capsule, and the posterior capsule is supplied by nerve to quadratus femoris and superior gluteal nerve (Laumonerie et al. 2021).

The PENGB is an interfascial plane block due to the local anesthetics is injected in the fascial plane between the psoas muscle and the superior pubic ramus (Alici et al. 2023). The clinical application of PENGB has gained increasing attention in hip surgeries. It has been successfully used for reducing acute traumatic pain of hip fractures (Lin et al. 2023), improving the pain during spinal anesthesia positioning (Erten et al. 2023) and providing effective analgesia for THA (Bravo et al. 2023). Previous conventional meta-analysis showed that the PENGB improved pain and reduced opioid consumption when compared with a control group (Ke et al. 2024; She et al. 2024; Pai et al. 2024). The main reason why PENGB can significantly relieve pain is that it blocks the articular branches of the FN and ON (Bravo et al. 2023), and these nerves supplies the anterior capsule which is the area of densest nociceptive innervation (Laumonerie 2021).

Theoretically, LPB is superior to other other regional analgesia techniques following THA because it can block the FN, obturator nerve, accessory obturator nerve, and LFCN (Bravo et al. 2020). But some clinical studies showed that LPB, FNB, and FICB had equivalent analgesic efficacy in hip surgeries (Li et al. 2022; Bravo et al. 2020). The FNB and FICB had common disadvantages of incomplete ON block (ONB) and leaded to quadriceps muscle weakness, which were harmful for early rehabilitation and recovery (Eshag et al. 2024). Thybo et al. did not find that LFCNB promoted the analgesic effect when combined with paracetamol and ibuprofen after THA (Thybo et al. 2016). Marty et al. did not find that alone ONB decrease postoperative opioid consumption after THA (Marty et al. 2021). These evidence indicates that FN is mainly responsible for the transmission of pain signals after THA, but the effect of LFCN and ON are secondary.

The QLB is an interfascial plane block, and it includes four approaches based on the relationship between the injection site and the quadratus lumbosus muscle. The posterior QLB can not reduce pain scores and morphine consumption after THA (Brixel et al. 2021), but the anterior QLB has an opposite effectiveness (Wang et al. 2022; Kukreja et al. 2019). Moreover, the lateral and transmuscular QLB can provide similar analgesia to LPB (Adhikary et al. 2018; Kelly et al. 2022).

The PLAI is required to infiltrate these tissues which includes anterior and posterior capsules, gluteus minimus and medius muscles, supraacetabular region, area around the anterior inferior iliac spine, the gluteus maximus muscle, iliotibial band, subcutaneous tissues, and skin (Bravo et al. 2023). Because it can not block any motor nerve, the rate of quadriceps weakness is lower than other regional nerves block. The PLAI can alleviate pain from the anterior capsule, the posterior capsule, the labrum, skin and others theoretically, so Bravo et al. found that the PLAI had lower static pain scores (especially during the first 24 h) and dynamic pain scores (first 6 h) than PENGB (Bravo et al. 2023). But the clinical effect of PLAI can be affected by drug dosage, drug volume, adjuvants, injection technique, and others (King et al. 2023). The ideal drug formula of PLAI has not been defined yet, it should be investigated by more high-quality studies in future (King et al. 2023). Based on the above review, PENGB can only alleviate pain from the anterior capsule, but PLAI can block the most nociceptor-rich region supplied by the articular branches of the FN and ON, and superior gluteal nerve. The PLAI can enhance the analgesic effect and compensate for the analgesic area of PENGB, so PENGB + PLAI is superior to PENGB or PLAI.

Our NMA has the following limitations. Firstly, three studies have sample sizes less than 60, which can easily lead to sampling errors and increase the risk of bias. Secondly, these differences are potential factors contributing to heterogeneity, which includes the type of anesthesia, the drugs given during regional analgesia, the rescuing analgesic regimens, and pain scales. Thirdly, our study focused on short-term (within 24 h) effectiveness of regional analgesia techniques and lacked analysis of data exceeded 24–48 h. Fifthly, the PONV and LOS were influenced by various factors such as the patient's comorbidities, surgical effect, anesthesia methods and anesthetics, and perioperative care and so on. So we should correctly interpret the impact of regional analgesia techniques on the PONV and LOS. Finally, with the publication of more high-quality RCTs with larger sample sizes, we need to reevaluated the ideal regional analgesia technique for THA and the optimal drug formula.

## Conclusions

Our systematic review and NMA shows that PENGB was found to have significantly lower incidence of quadriceps motor block and PONV. PENGB + PLAI was found to be significantly more effective than other treatments (PLAI, PENGB, QLB, FICB) in reducing resting and movement pain scores, opioid consumption within 24 h and the LOS.

## Supplementary Information

Supplementary Material 1.

## Data Availability

Data is provided within the manuscript and supplementary information files.

## Abbreviations

NMA: Network meta-analysis
RCTs: Randomized controlled trials
ERAS: Enhanced Recovery after Surgery
PONV: Postoperative nausea and vomiting
PCIA: Patient-controlled intravenous analgesia
THA: Total hip arthroplasty
PENGB: Pericapsular nerve group block
PLAI: Periarticular local anesthetic infiltration
QLB: Quadratus lumborum block
FICB: Fascia iliaca compartment block
LFCNB: Lateral femoral cutaneous nerve block
FNB: Femoral nerve block
LPB: Lumbar plexus block
SUCRA: Surface under the cumulative ranking curve
LOS: Length of hospital stay
