# Supplementary material for: Is pericapsular nerve group block superior to other regional analgesia techniques following total hip arthroplasty? a systematic review and network meta-analysis
Source: Perioper Med (Lond). 2024 Oct 4;13:96. doi: 10.1186/s13741-024-00455-y (PMC11451061; doi:10.1186/s13741-024-00455-y)
Supplement: Supplementary file 1 — Supplementary Material 1. [file 13741_2024_455_MOESM1_ESM.docx]

**Supplementary material**

**Title** - Is pericapsular nerve group block superior to other regional analgesia techniques following total hip arthroplasty? systematic review and network meta-analysis

**Contents**

[**Section 1. Network Geometry** **1**](#_Toc50023580)

[**Section 2. Network forest plots** **6**](#_Toc50023583)

[**Section 3. Exploration of inconsistency 11**](#_Toc50023584)

[**Section 4. Netleague tables of mixed estimates 14**](#_Toc50023585)

[**Section 5. SUCRA rankings 17**](#_Toc50023586)

[**Section 6. Subgroup analyses 22**](#_Toc50023589)

## Section 1. Network Geometry

**Resting VAS at 12 h after surgery**

The network was partly disconnected and control arm as the largest comparator.

|  |
| --- |
| Figure 1A. Network map of postoperative resting VAS at 12 h |

|  |
| --- |
| Figure 1B. Network map of postoperative resting VAS at 24 h |

|  |
| --- |
| Figure 1C. Network map of postoperative movement VAS at 6 h |

|  |
| --- |
| Figure 1D. Network map of postoperative movement VAS at 12 h |

|  |
| --- |
| Figure 1E. Network map of postoperative movement VAS at 24 h |

|  |
| --- |
| Figure 1F. Network map of postoperative opioid consumption within 24 h |

|  |
| --- |
| Figure 1G. Network map of PONV |

|  |
| --- |
| Figure 1H. Network map of quadriceps motor block |

|  |
| --- |
| Figure 1I. Network map of length of hospital stay |

## Section 2. Network forest plots

|  |
| --- |
| Figure 2A. Network forest plot of postoperative resting VAS at 12 h |

|  |
| --- |
| Figure 2B. Network forest plot of postoperative resting VAS at 24 h |

|  |
| --- |
| Figure 2C. Network forest plot of postoperative movement VAS at 6 h |

|  |
| --- |
| Figure 2D. Network forest plot of postoperative movement VAS at 12 h |

|  |
| --- |
| Figure 2E. Network forest plot of postoperative movement VAS at 24 h |

|  |
| --- |
| Figure 2F. Network forest plot of postoperative opioid consumption within 24 h |

|  |
| --- |
| Figure 2G. Network forest plot of PONV |

|  |
| --- |
| Figure 2H. Network forest plot of quadriceps motor block |

|  |
| --- |
| Figure 2I. Network forest plot of length of hospital stay |

## Section 3. Exploration of inconsistency

| Table 3A. Node-splitting of postoperative resting VAS at 12 h | | | | | | | |
| --- | --- | --- | --- | --- | --- | --- | --- |
| Side | Direct | | Indirect | | Difference | | P>\|z\| |
|  | Coef. | Std. Err. | Coef. | Std. Err. | Coef. | Std. Err. |  |
| FICB vs. PENG * | -.0936527 | .1574149 | .5340425 | 15.60961 | -.6276952 | 15.61041 | 0.968 |
| PENG vs. PLAI * | -.3255362 | .6145503 | .5221598 | 19.26699 | -.847696 | 19.27682 | 0.965 |
| PENG vs. QLB * | -.2399998 | .2344497 | .1871696 | 56.28725 | -.4271694 | 56.28812 | 0.994 |
| PENG+PLAI vs. PLAI* | .8699999 | .1377407 | -.8376699 | 38.92508 | 1.70767 | 38.92538 | 0.965 |
| * Warning: all the evidence about these contrasts comes from the trials which directly compare them. | | | | | | | |

| Table 3B. Node-splitting of postoperative resting VAS at 24 h | | | | | | | |
| --- | --- | --- | --- | --- | --- | --- | --- |
| Side | Direct | | Indirect | | Difference | | P>\|z\| |
|  | Coef. | Std. Err. | Coef. | Std. Err. | Coef. | Std. Err. |  |
| FICB vs. PENG * | -.514998 | .4987659 | .7151268 | 14.9336 | -1.230125 | 14.94191 | 0.934 |
| PENG vs. PLAI * | -.5403003 | .7727642 | .9269488 | 17.57692 | -1.467249 | 17.59397 | 0.934 |
| PENG vs. QLB * | -.2299999 | .7272157 | 1.027882 | 60.73313 | -1.257882 | 60.73763 | 0.983 |
| PENG+PLAI vs. PLAI* | .82 | .695294 | -2.110007 | 35.4851 | 2.930007 | 35.49196 | 0.934 |
| * Warning: all the evidence about these contrasts comes from the trials which directly compare them. | | | | | | | |

| Table 3C. Node-splitting of postoperative movement VAS at 6 h | | | | | | | | | | | | | | |  |
| --- | --- | --- | --- | --- | --- | --- | --- | --- | --- | --- | --- | --- | --- | --- | --- |
| Side | | Direct | | | Indirect | | | | Difference | | | | P>\|z\| | |  |
|  |  | Coef. | Std. Err. | | Coef. | | Std. Err. | | Coef. | | Std. Err. | |  |  |  |
| FICB vs. PENG * | | -2.548148 | .5551109 | | .4724239 | | 13.66628 | | -3.020572 | | 13.67755 | | 0.825 | |  |
| PENG vs. PLAI * | | -.7174674 | .2423607 | | 3.308142 | | 20.2474 | | -4.02561 | | 20.24886 | | 0.842 | |  |
| PENG vs. QLB * | | .5702688 | .151463 | | 5.053189 | | 42.01041 | | -4.48292 | | 42.0108 | | 0.915 | |  |
| PENG+PLAI vs. PLAI* | | 1.18 | .1511622 | | -6.526414 | | 42.38761 | | 7.706414 | | 42.38803 | | 0.856 | |  |
| * Warning: all the evidence about these contrasts comes from the trials which directly compare them. | | | | | | | | | | | | | | |  |
| Table 3D. Node-splitting of postoperative movement VAS at 12 h | | | | | | | | | | | | | | | |
| Side | Direct | | | | | Indirect | | | | Difference | | | | P>\|z\| | |
|  | Coef. | | | Std. Err. | | Coef. | | Std. Err. | | Coef. | | Std. Err. | |  |  |
| FICB vs. PENG * | .3032907 | | | .1873523 | | .7734638 | | 17.8438 | | -.4701731 | | 17.84478 | | 0.979 | |
| PENG vs. PLAI * | -.6940746 | | | .2451452 | | .0660658 | | 21.88817 | | -.7601404 | | 21.8896 | | 0.972 | |
| PENG vs. QLB * | -1.83e-11 | | | .3980369 | | -.6066944 | | 97.40818 | | .6066944 | | 97.40884 | | 0.995 | |
| PENG+PLAI vs. PLAI* | .8899999 | | | .1658011 | | .1658011 | | -.7812754 | | 46.18847 | | 1.671275 | | 0.971 | |
| * Warning: all the evidence about these contrasts comes from the trials which directly compare them. | | | | | | | | | | | | | | | |

| Table 3E. Node-splitting of postoperative movement VAS at 24 h | | | | | | | |
| --- | --- | --- | --- | --- | --- | --- | --- |
| Side | Direct | | Indirect | | Difference | | P>\|z\| |
|  | Coef. | Std. Err. | Coef. | Std. Err. | Coef. | Std. Err. |  |
| FICB vs. PENG * | -1.035612 | .6907112 | .3554545 | 15.86188 | -1.391066 | 15.87692 | 0.930 |
| PENG vs. PLAI * | .0999989 | 1.947868 | 1.565988 | 17.88876 | -1.465989 | 17.99446 | 0.935 |
| PENG vs. QLB * | -.2 | .9744084 | 2.067942 | 68.0181 | -2.267942 | 68.02509 | 0.973 |
| PENG+PLAI vs. PLAI* | 1.06 | .9464459 | -1.870029 | 36.00893 | 2.930029 | 36.02148 | 0.935 |
| * Warning: all the evidence about these contrasts comes from the trials which directly compare them. | | | | | | | |

| Table 3F. Node-splitting of postoperative opioid consumption within 24 h | | | | | | | |
| --- | --- | --- | --- | --- | --- | --- | --- |
| Side | Direct | | Indirect | | Difference | | P>\|z\| |
|  | Coef. | Std. Err. | Coef. | Std. Err. | Coef. | Std. Err. |  |
| FICB vs. PENG * | -13.34664 | 8.301001 | .7639508 | 93.52654 | -14.11059 | 93.89474 | 0.881 |
| PENG vs. PLAI * | .3329935 | 6.046933 | 21.4302 | 142.5384 | -21.09721 | 142.667 | 0.882 |
| PENG+PLAI vs. PLAI* | 8.119485 | 8.544806 | -25.82361 | 378.9499 | 33.94309 | 379.0495 | 0.929 |
| * Warning: all the evidence about these contrasts comes from the trials which directly compare them. | | | | | | | |

| Table 3G. Node-splitting of PONV | | | | | | | |
| --- | --- | --- | --- | --- | --- | --- | --- |
| Side | Direct | | Indirect | | Difference | | P>\|z\| |
|  | Coef. | Std. Err. | Coef. | Std. Err. | Coef. | Std. Err. |  |
| FICB vs. PENG * | -.1188776 | .514335 | -.1576315 | 33.58255 | .0387539 | 33.58543 | 0.999 |
| PENG vs. PLAI * | .237326 | .4265935 | .0895324 | 58.1799 | .1477936 | 58.18642 | 0.998 |
| PENG vs. QLB * | .1801292 | .4261771 | .2321033 | 122.4867 | -.0519741 | 122.4891 | 1.000 |
| PENG+PLAI vs. PLAI* | -.2673622 | .5185114 | .266132 | 149.8217 | -.5334942 | 149.8228 | 0.997 |
| * Warning: all the evidence about these contrasts comes from the trials which directly compare them. | | | | | | | |

| Table 3H. Node-splitting of quadriceps motor block | | | | | | | |
| --- | --- | --- | --- | --- | --- | --- | --- |
| Side | Direct | | Indirect | | Difference | | P>\|z\| |
|  | Coef. | Std. Err. | Coef. | Std. Err. | Coef. | Std. Err. |  |
| FICB vs. PENG * | -2.771943 | .7503888 | -.2059211 | 51.63465 | -2.566022 | 51.64011 | 0.960 |
| PENG vs. PLAI * | 5.32e-10 | 2.016327 | 2.736243 | 75.88044 | -2.736243 | 75.90722 | 0.971 |
| PENG vs. QLB * | .6937381 | .6024485 | 5.603343 | 145.2182 | -4.909605 | 145.219 | 0.973 |
| PENG+PLAI vs. PLAI* | -.1967103 | .6282267 | -5.537831 | 153.5548 | 5.34112 | 153.5563 | 0.972 |
| * Warning: all the evidence about these contrasts comes from the trials which directly compare them. | | | | | | | |

| Table 3I. Node-splitting of length of hospital stay | | | | | | | |
| --- | --- | --- | --- | --- | --- | --- | --- |
| Side | Direct | | Indirect | | Difference | | P>\|z\| |
|  | Coef. | Std. Err. | Coef. | Std. Err. | Coef. | Std. Err. |  |
| FICB vs. PENG * | .0132304 | .1548909 | .065153 | 10.02426 | -.0519226 | 10.02546 | 0.996 |
| PENG vs. PLAI * | .0050093 | .0966402 | .1015431 | 13.03758 | -.0965337 | 13.03795 | 0.994 |
| PENG+PLAI vs. PLAI* | .3099999 | .1066068 | .0364875 | 29.72252 | .2735124 | 29.72284 | 0.993 |
| * Warning: all the evidence about these contrasts comes from the trials which directly compare them. | | | | | | | |

**Section 4. Netleague tables of mixed estimates**

| Table 4A. Netleague of postoperative resting VAS at 12 h | | | | |
| --- | --- | --- | --- | --- |
|  | QLB | PLAI | PENG+PLAI | PENG |
| FICB | -0.33 (-0.89,0.22) | -0.42 (-1.66,0.82) | -1.29 (-2.56,-0.02) | -0.09 (-0.40,0.21) |
|  | QLB | -0.08 (-1.37,1.20) | -0.95 (-2.27,0.36) | 0.24 (-0.22,0.70) |
|  |  | PLAI | -0.87 (-1.14,-0.60) | 0.32 (-0.88,1.53) |
|  |  |  | PENG+PLAI | 1.19 (-0.04,2.43) |
|  |  |  |  | PENG |

| Table 4B. Netleague of postoperative resting VAS at 24 h | | | | |
| --- | --- | --- | --- | --- |
|  | QLB | PLAI | PENG+PLAI | PENG |
| FICB | -0.74 (-2.47,0.98) | -1.05 (-2.85,0.75) | -1.87 (-4.13,0.38) | -0.51 (-1.49,0.46) |
|  | QLB | -0.31 (-2.39,1.77) | -1.13 (-3.61,1.36) | 0.23 (-1.20,1.65) |
|  |  | PLAI | -0.82 (-2.18,0.54) | 0.54 (-0.98,2.05) |
|  |  |  | PENG+PLAI | 1.36 (-0.68,3.39) |
|  |  |  |  | PENG |

| Table 4C. Netleague of postoperative movement VAS at 6 h | | | | |
| --- | --- | --- | --- | --- |
|  | QLB | PLAI | PENG+PLAI | PENG |
| FICB | -1.97 (-3.10,-0.85) | -3.26 (-4.45,-2.07) | -4.44 (-5.66,-3.22) | -2.54 (-3.63,-1.46) |
|  | QLB | -1.29 (-1.85,-0.73) | -2.47 (-3.10,-1.83) | -0.57 (-0.87,-0.27) |
|  |  | PLAI | -1.18 (-1.48,-0.88) | 0.72 (0.24,1.19) |
|  |  |  | PENG+PLAI | 1.90 (1.34,2.46) |
|  |  |  |  | PENG |

| Table 4D. Netleague of postoperative movement VAS at 12 h | | | | |
| --- | --- | --- | --- | --- |
|  | QLB | PLAI | PENG+PLAI | PENG |
| FICB | 0.30 (-0.56,1.17) | -0.39 (-1.00,0.21) | -1.28 (-1.97,-0.59) | 0.30 (-0.06,0.67) |
|  | QLB | -0.69 (-1.61,0.22) | -1.58 (-2.56,-0.61) | 0.00 (-0.78,0.78) |
|  |  | PLAI | -0.89 (-1.21,-0.57) | 0.69 (0.21,1.17) |
|  |  |  | PENG+PLAI | 1.58 (1.00,2.16) |
|  |  |  |  | PENG |

| Table 4E. Netleague of postoperative movement VAS at 24 h | | | | |
| --- | --- | --- | --- | --- |
|  | QLB | PLAI | PENG+PLAI | PENG |
| FICB | -1.23 (-3.57,1.10) | -0.92 (-4.94,3.10) | -1.97 (-6.39,2.44) | -1.03 (-2.38,0.32) |
|  | QLB | 0.32 (-3.93,4.56) | -0.74 (-5.37,3.88) | 0.20 (-1.71,2.11) |
|  |  | PLAI | -1.06 (-2.90,0.79) | -0.12 (-3.91,3.67) |
|  |  |  | PENG+PLAI | 0.94 (-3.27,5.15) |
|  |  |  |  | PENG |

| Table 4F. Netleague of postoperative opioid consumption within 24 h | | | |
| --- | --- | --- | --- |
|  | PLAI | PENG+PLAI | PENG |
| FICB | -12.86 (-32.80,7.08) | -20.96 (-47.02,5.10) | -13.23 (-29.36,2.90) |
|  | PLAI | -8.10 (-24.82,8.62) | -0.37 (-12.17,11.43) |
|  |  | PENG+PLAI | 7.73 (-12.73,28.19) |
|  |  |  | PENG |

| Table 4G. Netleague of PONV | | | | | |
| --- | --- | --- | --- | --- | --- |
|  | QLB | PLAI | PENG+PLAI | PENG+LFCN | PENG |
| FICB | 0.06 (-1.25,1.37) | 0.12 (-1.17,1.41) | 0.39 (-1.26,2.03) | -0.00 (-2.00,2.00) | -0.12 (-1.13,0.89) |
|  | QLB | 0.06 (-1.13,1.25) | 0.32 (-1.24,1.89) | -0.06 (-2.46,2.33) | -0.18 (-1.02,0.66) |
|  |  | PLAI | 0.27 (-0.75,1.28) | -0.12 (-2.50,2.26) | -0.24 (-1.06,0.59) |
|  |  |  | PENG+PLAI | -0.39 (-2.98,2.20) | -0.50 (-1.81,0.80) |
|  |  |  |  | PENG+LFCN | -0.12 (-2.36,2.12) |
|  |  |  |  |  | PENG |

| Table 4H. Netleague of quadriceps motor block | | | | |
| --- | --- | --- | --- | --- |
|  | QLB | PLAI | PENG+PLAI | PENG |
| FICB | -2.08 (-3.96,-0.19) | -2.77 (-6.98,1.45) | -2.57 (-6.96,1.82) | -2.77 (-4.24,-1.30) |
|  | QLB | -0.69 (-4.82,3.43) | -0.50 (-4.80,3.81) | -0.69 (-1.87,0.49) |
|  |  | PLAI | 0.20 (-1.03,1.43) | -0.00 (-3.95,3.95) |
|  |  |  | PENG+PLAI | -0.20 (-4.34,3.94) |
|  |  |  |  | PENG |

| Table 4I. Netleague of length of hospital stay | | | |
| --- | --- | --- | --- |
|  | PLAI | PENG+PLAI | PENG |
| FICB | 0.02 (-0.34,0.38) | -0.29 (-0.71,0.12) | 0.01 (-0.29,0.32) |
|  | PLAI | -0.31 (-0.52,-0.10) | -0.01 (-0.19,0.18) |
|  |  | PENG+PLAI | 0.30 (0.02,0.59) |
|  |  |  | PENG |

## Section 5. SUCRA rankings

|  |
| --- |
| Figure 5A. SUCRA of postoperative resting VAS at 12 h. 1=FICB(82.8), 2=PENG(70.3), 3=PENG+PLAI(3.3), 4=PLAI(50.7), 5=QLB(42.9) |

|  |
| --- |
| Figure 5B. SUCRA of postoperative resting VAS at 24 h. 1=FICB(84.6), 2=PENG(60.9), 3=PENG+PLAI(11.2), 4=PLAI(40.4), 5=QLB(52.7 ) |

|  |
| --- |
| Figure 5C. SUCRA of postoperative movement VAS at 6 h. 1=FICB(100.0), 2=PENG(49.9), 3=PENG+PLAI(0.0), 4=PLAI(25.1), 5=QLB(75.0) |

|  |
| --- |
| Figure 5D. SUCRA of postoperative movement VAS at 12 h. 1=FICB(54.6), 2=PENG(85.6), 3=PENG+PLAI(0.0), 4=PLAI(29.1), 5=QLB(80.6 ) |

|  |
| --- |
| Figure 5E. SUCRA of postoperative movement VAS at 24 h. 1=FICB(80.3), 2=PENG(44.0), 3=PENG+PLAI(27.0), 4=PLAI(59.2), 5=QLB(39.5) |
|  |
| Figure 5F. SUCRA of postoperative opioid consumption within 24 h. 1=FICB(92.3), 2=PENG(44.1), 3=PENG+PLAI(15.1), 4=PLAI(48.6) |

|  |
| --- |
| Figure 5G. SUCRA of PONV. 1=FICB(45.5), 2=PENG(34.7), 3=PENG+LFCN(49.2), 4=PENG+PLAI(68.1), 5=PLAI(52.2), 6=QLB(50.3) |

|  |
| --- |
| Figure 5H. SUCRA of quadriceps motor block. 1=FICB(93.3), 2=PENG(26.8), 3=PENG+PLAI(42.9), 4=PLAI(33.8), 5=QLB(53.2) |

|  |
| --- |
| Figure 5I. SUCRA of length of hospital stay. 1=FICB(61.6), 2=PENG(68.0), 3=PENG+PLAI(3.0), 4=PLAI(67.5) |

**Section 6. Subgroup analyses**

**Type of anesthesia (spinal anesthesia)**

|  |
| --- |
| Figure 6A. Network map of spinal anesthesia |

|  |
| --- |
| Figure 6B. SUCRA of spinal anesthesia.1= FICB(82.5), 2=PENG(47.2), 3=PLAI(3.4), 4=QLB(66.8) |

|  |
| --- |
| Figure 6C. Network forest plot of of spinal anesthesia |

| Table 6A. Node-splitting of spinal anesthesia | | | | | | | |
| --- | --- | --- | --- | --- | --- | --- | --- |
| Side | Direct | | Indirect | | Difference | | P>\|z\| |
|  | Coef. | Std. Err. | Coef. | Std. Err. | Coef. | Std. Err. |  |
| FICB vs. PENG * | -.1963429 | .1576977 | .0386339 | 26.04225 | -.2349769 | 26.04273 | 0.993 |
| PENG vs. PLAI * | -1.022376 | .5752681 | .4198075 | 132.436 | -1.442183 | 132.4376 | 0.991 |
| PENG vs. QLB * | .0999999 | .2311781 | .3926825 | 56.59638 | -.2926826 | 56.59691 | 0.996 |
| * Warning: all the evidence about these contrasts comes from the trials which directly compare them. | | | | | | | |

| Table 6B. Netleague of spinal anesthesia | | | |
| --- | --- | --- | --- |
|  | QLB | PLAI | PENG |
| FICB | -0.10 (-0.64,0.45) | -1.22 (-2.39,-0.05) | -0.20 (-0.51,0.11) |
|  | QLB | -1.12 (-2.34,0.09) | -0.10 (-0.55,0.35) |
|  |  | PLAI | 1.02 (-0.11,2.15) |
|  |  |  | PENG |

|  |
| --- |
| Figure 6D. Funnel plot of spinal anesthesia |

**Type of anesthesia (general anesthesia)**

|  |
| --- |
| Figure 6E. Network map of general anesthesia |
